# Survey of the Mosquitoes (Diptera: Culicidae) of Mayotte

**DOI:** 10.1371/journal.pone.0100696

**Published:** 2014-07-08

**Authors:** Gilbert Le Goff, Steven M. Goodman, Eric Elguero, Vincent Robert

**Affiliations:** 1 Maladies Infectieuses et Vecteurs: Ecologie, Génétique, Evolution et Contrôle (IRD 224, CNRS 5290, Université de Montpellier), Institut de Recherche pour le Développement, Montpellier, France; 2 Field Museum of Natural History, Chicago, Illinois, United States of America; 3 Association Vahatra, Antananarivo, Madagascar; Federal University of Viçosa, Brazil

## Abstract

A transversal survey of immature mosquitoes was conducted on Mayotte Island (France) in the Comoros Archipelago, western Indian Ocean, with the aim to inventory the Culicidae and to document inter-species relationships in different habitats. In total 420 habitats were sampled for larvae and/or pupae mosquitoes, resulting in more than 6,000 specimens. Forty species belonging to 15 genera were collected, with eight taxa integrated for the first time to the Mayotte mosquito list. The most frequently recorded species were *Stegomyia aegypti*, *St. albopicta*, *Anopheles gambiae* and *Eretmapodites subsimplicipes*, the first three species being known vectors of viruses and parasites transmitted to humans. Mean species richness in habitats ranged from 1.00 to 3.29, with notable differences between habitats. For example, water-filled axils of banana leaves, tree-holes and crab-holes had low species richness, while cut bamboo, water pools, abandoned tires and marsh and swamp water had notably higher species richness. Twenty-seven mosquito species belonging to 12 genera were routinely collected (in ≥20% of at least one type of larval habitat) suggesting that multiple species play a role in the biocenosis of these aquatic habitats. Multispecies association was observed in 52% of the habitats. The co-occurrence of up to six species belonging to five genera was recorded in a single habitat. The mosquitoes of Mayotte show notable biogeographical affinities to those of Madagascar, as compared to the African continent. These two potential source areas are nearly equidistant from Mayotte, which in turn indicates biased dispersal from east to west. Our findings suggest that with relatively short-term intensive sampling in different habitats, it is possible to approach exhaustive species inventories based on collection of larvae. Mayotte, with its modest elevation range and land surface, has a notable species richness of mosquitoes with 45 well-documented species belonging to 15 genera.

## Introduction

The past decades have seen the emergence of large-scale biodiversity studies and biological inventories of poorly or completely unknown areas of Earth. Because of their lack of exploration and high levels of taxonomic diversity, different areas in the tropics have been the foci of a large number of such investigations. In many cases, these field surveys are disproportionately focused on vertebrates, as compared to invertebrates, with respect to the taxonomic representation of these two groups within a given ecosystem. Starting with some pioneering work of entomologists such as Terry L. Erwin and Nigel Stork, as well as others, different field and laboratory techniques have been developed to sample, handle and identify the myriads of specimens that arise from invertebrate surveys in the tropics, e.g. [1]–[5]. Recently, based on the tenebrionid beetles of the Latium Region of Italy, Simone Fattorini [6] underlined the considerable cost effective source of knowledge derived from collections made by amateur naturalists and the importance of such museological material for faunistic studies in poorly surveyed areas. The level of discovery of undescribed taxa during the course of these surveys is staggering, which in turn leads to the ongoing question as to how many arthropods exist in the world and what proportion are unknown to science [4], [7]–[12].

Another important phase in the documentation of arthropods and other poorly studied plant and animal groups in the tropics in the context of broad-scale surveys was Daniel Janzen's proposition for “All-Taxa Biological Inventory” (ATBI) programs (e.g. [13], [14]); the intent was to document and identify all biological species for a given group of organisms in a delineated geographical area. This concept in various forms was adapted to different portions of the world to document existing biodiversity [15]–[18]. ATBIs conducted in areas with considerable surface area and ecological heterogeneity are inherently incomplete since even well studied areas hold numerous taxa new to science and involve a dynamic process of immigration-extinction from neighbouring areas. Hence, for these reasons, ATBI style inventories on tropical oceanic islands, particularly those formed *de novo* and with relatively limited surface area, are highly appropriate for surveys that attempt to approach complete documentation for a given biotic group.

Herein we report a survey of mosquitoes conducted on Mayotte, an *in situ* volcanic island part of the Comoros Archipelago, in the context of an ATBI. This survey was aimed at updating a mosquito inventory carried out on Mayotte by Jacques Brunhes about 30 years ago [19]. Our work helps to complete the survey of mosquitoes recently performed on other islands in the Comoros Archipelago, namely Grande Comore, Anjouan and Mohéli [20]. This later survey focused on population genetics of Anopheline mosquitoes, but opportunistic collections of larva were made from pools of standing water such as near roads and in rice fields or swamps within or near villages. Our field survey was not limited to village settings and covered numerous zones and natural ecosystems of Mayotte. The inventory concentrated on obtaining larvae and pupae from a variety of different habitats. Given the intensity and completeness of this survey, including detailed morphological and molecular genetic identification of taxa, meaningful inferences can be made on ecological associations between species (intrageneric and intergeneric) and the biogeography (dispersal history) of the local Culicidae. As different mosquitoes on the islands in the Comoros Archipelago are known vectors of human and zoonotic disease [19]–[36], the obtained faunistic data also provide important insight from a public health perspective.

## Materials and Methods

### Study area

Mayotte is an Overseas Department and Region of France in the western Indian Ocean and consists of the main island (Grande-Terre), a smaller island (Petite-Terre), and several islets. This archipelago is located in the northern Mozambique Channel, 250 km W of Madagascar and 450 km E of Mozambique. Mayotte is geographically part of the Comoro Archipelago, but has been politically separate since 1975 from three other islands (Grande Comore, Mohéli and Anjouan), which form the Union of the Comoros. The nearest island to Mayotte in the archipelago is Anjouan, 30 km to the NNE.

Mayotte has a surface area of 374 km^2^, mostly comprising Grande-Terre (363 km^2^), which is 39 km long and 22 km wide, and rising to 660 m above sea level (Mount Benara). Mamoudzou is the largest city on the island, and serves as the harbour and administrative centre. Estimates of residing human populations show a dramatic increase from about 23,364 inhabitants in 1958, 186,452 censused in July 2007 and an estimated 212,000 people in 2012 [37]. Mayotte has a population density of 567 individuals per km^2^.

### Entomological study

**Figure 1:**
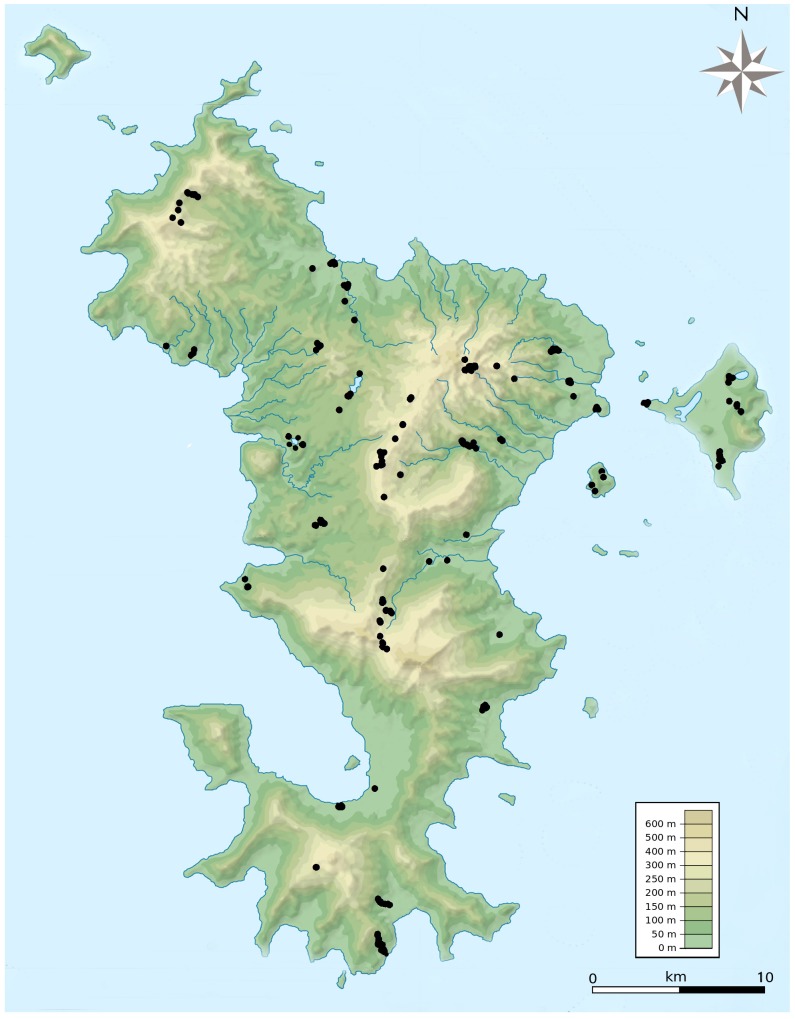
Prospecting effort associated with larval mosquito inventories on Mayotte during the 2008–2012 surveys. In total, 420 habitats yielded mosquito collections and in certain cases, theseare in close proximity and overlap as a single point on the map.

Our mosquito research was mainly focused on aquatic preimaginal stages. Natural areas were given priority due to anticipated higher specific richness in these areas, although urban and agricultural zones were also surveyed. Further justification for concentrating on non-urban areas is that Bagny and colleagues [34] performed Aedini mosquito surveys in six urban localities on Mayotte in 2007 and data and specimens from such sites are already available. In chronological order, field collections were made (season and number of larval habitats in parentheses) on 12 October 2008 (dry season, 2 habitats), from 22 March to 6 April 2011 (end of rainy season, 366), from 22 November to 2 December 2011 (start of rainy season, 50), and from 13 to 19 November 2012 (start of rainy season, 2). No individual habitat was sampled more than once. All habitat types were systematically examined during the field surveys, with the exception of those with numerous abandoned solid wastes (see below “Description of the larval habitats”) that were limited to five habitats of this type at each site. Censused habitats without larvae or pupae of Culicidae were not considered in the present study. In total 420 habitats yielded mosquito collections and are analysed herein. The habitat locations are given in Figure 1. The field study did not involve endangered or protected species and no specific permit was required for collecting mosquitoes in these locations. Larvae and pupae of mosquitoes were obtained from habitats by use of small dip nets and removal of water with pipettes; in many cases the contents were emptied into a white dish to allow more detailed examination [38]. Larvae were fixed in 70% ethanol. Pupae were reared in small mesh cages resting in freshwater to obtain adult male and female mosquitoes after their emergence. No special method was used for collecting *Mansonia* larvae, which are generally submerged and anchored by the siphon to aquatic plant roots, including those of introduced *Pistia*. Our mosquito specimens are deposited in the Collection d'Arthropodes d'Intérêt Médical (ARIM), Laboratoire de Taxonomie des Vecteurs, Institut de Recherche pour le Développement, Montpellier, France. We have also examined other material collected in the Comoros Archipelago and Madagascar housed at ARIM.

### Mosquito identification to species level

Identification was mainly based on morphological characters, both for larvae and adults [39]–[55]. One exception includes mosquitoes of the *Anopheles gambiae* complex, for which we relied on molecular sequencing for species identification [56]. A second exception concerns specimens of *Stegomyia* of the subgenus *Mukwaya*, for which *St. bromeliae* was the only species previously reported on Mayotte (see [57]). For these mosquitoes, both morphological and gene sequencing characters were used.

We have adopted the mosquito classification and taxonomy proposed by Reinert and collaborators [58] and Harbach [59]. Because generic allocation of certain mosquito species has been the subject of some taxonomic flux, earlier classification of the tribe Aedini used for Mayotte mosquitoes is presented in Table 1. Abbreviations for genera are those of Reinert [60]. We employ the morphological terminology of Harbach and Knight [61], [62].

**Table 1:** Taxonomic synonymies from different published sources of mosquito species occurring on Mayotte and belonging to the tribe Aedini.

Harbach (2014) [59]	Older sources
*Polyleptiomyia albocephala*	*Aedes* (*Aedes*) *albocephalus*
*Aedimorphus fowleri*	*Aedes* (*Aedimorphus*) *fowleri*
*Neomelaniconion circumluteolus*	*Aedes* (*Neomelaniconion*) *circumluteolus*
*Fredwardsius vittatus*	*Aedes* (*Fredwardsius*) *vittatus*
*Stegomyia* (*Stegomyia*) *aegypti*	*Aedes* (*Stegomyia*) *aegypti*
*Stegomyia albopicta*	*Aedes* (*Stegomyia*) *albopictus*
*Stegomyia* (*Mukwaya*) *bromeliae*	*Aedes* (*Stegomyia*) *bromeliae*
*Stegomyia* (*Stegomyia*) *pia*	*Aedes* (*Stegomyia*) *pia*
*Zavortinkius brunhesi*	*Aedes* (*Zavortinkius*) *brunhesi*
*Zavortinkius monetus*	*Aedes* (*Zavortinkius*) *monetus*
*Skusea cartroni*	*Aedes* (*Skusea*) *cartroni*
**7 genera**	**1 genus**

### Description of the larval habitats

Amongst a notable variety of habitats that retain water and where aquatic stages of mosquitoes can develop, we defined 20 specific habitat types (Table 2). These habitat types are grouped as follows.

**Table 2:** Mean species and genera richness in the different habitat types of Mayotte mosquitoes.

Type of habitat	n	Mean species richness	Standard deviation	Mean genus richness	Standard deviation	Significant species (number of habitats)
Snail shells	1	1.00	-	1.00	-	*Er. quinquevittatus* (1)
Axils of banana leaves	7	1.14	0.38	1.14	0.38	*St. bromeliae* (7)
Tree-holes	70	1.43	0.77	1.24	0.49	*Za. monetus* gr. (28), *Or. comorensis* (24)
Axils of pineapple plants	7	1.43	0.53	1.29	0.49	*St. bromeliae* (7)
Crab-holes	7	1.43	0.53	1.43	0.53	*Sk. cartroni* (6)
Holes in rock	27	1.44	0.64	1.19	0.40	*St. aegypti* (7), *Fr. vittatus* (5), *St. pia* (5)
Slow flowing water	22	1.50	0.60	1.23	0.43	*An. coustani* (11), *An. gambiae* (6), *Ur. mayottensis* (3)
Fallen leaf litter	26	1.61	0.94	1.54	0.71	*Er. subsimplicipes* (16), *Ur. comorensis* (14), *St. aegypti* (6)
Disposed solid waste	47	1.68	0.84	1.32	0.52	*St. albopicta* (24), *St. aegypti* (16), *Er. subsimplicipes* (10)
Coconut husk	16	1.69	1.08	1.38	0.50	*Er. subsimplicipes* (11), *St. aegypti* (6), *Cx. nebulosus* (3)
Axils of taro plants	4	1.75	0.50	1.75	0.50	*St. bromeliae* (4), *Mi. grjebinei* (3)
Large artificial container	14	1.79	0.70	1.57	0.65	*St. aegypti* (5), *Lu. tigripes* (4), *St. albopicta* (3), *Cx. quinquefasciatus* (3)
Holes in ground	8	2.00	0.76	1.63	0.52	*St. albopicta* (2), *Sk. cartroni* (2), *Am. fowleri* (2), *An. coustani* (2), C*x. wigglesworthi* (2)
Mushroom caps	4	2.00	0.82	1.75	0.50	*Ur. comorensis* (3), *Er. subsimplicipes* (3)
Water ponds and pools	67	2.04	1.09	1.66	0.81	*An. gambiae* (42), *Cx. simpsoni* (15), *Am. fowleri* (13)
Cut bamboo	45	2.11	0.98	1.80	0.81	*St. aegypti* (13), *Er. subsimplicipes* (12), *St. pia* (11), *Or. comorensis* (10), *St. albopicta* (9), *Cx. carleti* (9), *Cx. horridus* (9)
Vegetable and plant matter	9	2.22	1.20	2.00	0.87	*Er. subsimplicipes* (4), *Ur. comorensis* (4), *St. aegypti* (3)
Axils of *Typhonodorum* plants	17	2.24	0.75	2.06	0.75	*St. bromeliae* (17), *Mi. grjebinei* (11), *Ur. laffosseae* (5)
Discarded tires	5	2.80	1.48	2.20	1.64	*St. albopicta* (3), *St. aegypti* (2), *Lu. tigripes* (2), *Cx. decens* (2)
Marsh and swamp water	17	3.29	1.45	1.94	0.66	*An. coustani* (11), *An. gambiae* (11), *Cx. simpsoni* (8), *Cx. antennatus* (6), *Cx. tritaeniorhynchus* (4)
Total & mean	420	1.82	0.99	1.52	0.70	27 species belonging to 12 genera

The habitat types are listed by increasing species richness rank. The most frequent species are recorded as significant if present in more than 20% of samples for a larval habitat type.

#### Natural containers of vegetal origin that hold water (phytotelmata)

Immature stages of phytotelma-living mosquitoes frequently bred in the axils of different plants, such as introduced banana (1), pineapple (2), taro (Araceae, genus *Colocasia*) (3) and native *Typhonodorum lindleyanum* (Araceae) (4). Other phytotelmata include tree-holes (5) or internal sections of bamboo (6), which had been opened in most cases after being cut; leaf litter on the ground (7) often retains water pockets, and include, for example, dense coverage of fallen mango tree leaves or coconut palm fronds. Other phytotelmata include coconut husks (8), whether opened by human or with holes associated with rat damage; mushroom caps (9), which may hold small quantities of rain water; or different types of vegetable and plant matter (10) including cacao pods, leaves and pockets in different types of trees and fallen vegetational parts.

#### Natural containers of animal origin

Shells of introduced and invasive *Achatina* snails (Mollusca: Achatinidae) (11) were the only habitat of animal origin included herein.

#### Pools of water of different size and origin

Crab-holes (12) in the ground occurred in high density near mangrove areas; holes in the ground (13) of seemingly natural appearance were usually associated with human agricultural activities; and natural water pools in rock holes (14), often of volcanic origin. Some larval habitats were associated with natural water sources, such as slow moving sections of rivers (15) and ponds and pools (16) that are subject to completely drying-out. By contrast, marsh and swamp areas (17) provided permanent/subpermanent stagnant water.

#### Artificial human-made containers

These include abandoned solid waste (18), such as bottles, cans, plastic and metal pieces, often of discarded cars. Finally, tires (19) and larger artificial objects (20), often abandoned, such as boats, refrigerators, tubs, or barrels and tanks completed our defined habitat types.

This habitat typology is primarily based on the nature of the recipient, but the associated water sources, also vary from rain (natural containers), river (marsh and swamp), sea and brackish water (crab-holes in mangrove), or deliberately stocked or filled based on different human activities (tanks).

### Species accumulation curve and data analysis

A species accumulation curve derived as a plot of cumulative number of species discovered as a function of research effort [63]. Each species is considered regardless of its abundance or rarity. To take into account sampling error and the habitat heterogeneity among the habitats sampled, the order of the 420 habitats was randomized 30 times. The mean and standard deviation were computed, which became stable after about 20 randomizations. Mean species richness in a habitat type is the arithmetic mean of all the taxa identified at a given habitat.

The interaction between categorical variables ‘larval habitat types’ and ‘mosquito species’ were analysed through a multivariate statistical technique known as correspondence analysis [64], which provides visual representation in two-dimensional graphical form.

Mosquito species association was tested for each species pair and each habitat type, in cases that both members of the species pair were recorded together in at least 10 collection places of the same habitat. We used Fisher's exact test of independence with Bonferroni's correction.

## Results and Discussion

### The 2008–2012 surveys

During our inventories of Mayotte mosquitoes, 420 habitats with at least one larva or pupa were located across the island (Fig. 1). A few habitat types accounted for the majority of the collections: 48.8% for natural containers of vegetal origin, 35.2% for pools of water of different size and origin, 15.7% for artificial human-made containers and 0.3% for natural containers of animal origin. Details per type of habitat are presented in Table A in File S1. These collections resulted in more than 6,000 specimens, which represented 40 species belonging to 15 genera (Table 3). Thirty-three species of mosquitoes were previously reported on the island.

**Table 3:** Comprehensive list of the 50 mosquito species (Diptera: Culicidae) reported at least once on Mayotte based on the earliest records for the island in the beginning of the 20th-century to 2012.

Sub-family	Genus	(Subgenus)	Species, and subspecies if any	Species descriptor and year of description	First mention on Mayotte	First mention in the Comoros (other than Mayotte)	Specimens collected during the 2008–2012 surveys of Mayotte	Area of distribution (with references mentioning the absence on Mayotte)
*Anophelinae*	*Anopheles*	(*Anopheles*)	*coustani*	Laveran, 1900	[94]	[68] as *An. mauritianus*	yes	Tropical Africa, Madagascar, La Réunion, Mauritius, Mayotte, Anjouan, Mohéli
		(*Cellia*)	*comorensis*	Brunhes, Le Goff & Geoffroy, 1997	[95]		no	Endemic to Mayotte
			*funestus*	Giles, 1900	[94]	[68]	yes	Tropical Africa, Madagascar, Mayotte, Anjouan, Mohéli
			*gambiae s.s.*	Giles, 1902	[94] ecological identification; [74] molecular identification	[68] as *An. costalis*; [71] chromosomal and electrophoretic identification	yes	Tropical Africa, Madagascar, Comoros Archipelago
			*maculipalpis*	Giles, 1902	[70]	[46]	no	Tropical Africa, Madagascar, Mauritius, Mayotte, Mohéli
			*mascarensis*	de Meillon, 1947	[70]	[46]	yes	Madagascar, Mayotte, Anjouan, Mohéli
			*merus*	Dönitz, 1902	[70] morphological identification; this article with molecular identification	[46] ecological identification	yes	East and South Africa, Madagascar, Mauritius, Comoros Archipelago (ecological identification)
			*pretoriensis*	(Theobald, 1903)	[94]		yes	Tropical Africa, Madagascar, Comoros Archipelago
*Culicinae*	*Aedimorphus*		*fowleri*	(de Charmoy, 1908)	[47]		yes	Tropical Africa, Madagascar, La Réunion, Mauritius, Grande Comore, Mayotte
	*Culex*	(*Culex*)	*antennatus*	(Becker, 1903)	[47]		yes	Middle East, Africa, Madagascar, Seychelles
		(*Culex*)	*carleti*	Brunhes & Ravaonjanahary, 1971	[47]		yes	Madagascar
		(*Culex*)	*comorensis comorensis*	Brunhes, 1977	[47]		yes	Madagascar, Comoros Archipelago
		(*Culex*)	*decens*	Theobald, 1901	[47]		yes	Ethiopian Region, Madagascar, Comoros Archipelago (Mayotte, Anjouan)
		(*Culex*)	*quinquefasciatus*	Say, 1823	[70]	[94] as *Culex pipiens fatigans*	yes	Pantropical
		(*Culex*)	*simpsoni*	Theobald, 1905	[47]	[94]	yes	Tropical Africa, Madagascar, Seychelles, Comoros Archipelago
		(*Culex*)	*tritaeniorhynchus*	Giles, 1901	This article		yes	Asia, Middle East, Africa, Madagascar, La Réunion, Seychelles, Mayotte
		(*Culiciomyia*)	*cinerellus*	Edwards, 1922	[47]		yes	Tropical Africa, Madagascar, Mayotte, Mohéli
		(*Culiciomyia*)	*nebulosus nebulosus*	Theobald, 1901	[47]		yes	Tropical Africa, Madagascar, Mayotte
		(*Eumelanomyia*)	*horridus horridus*	Edwards, 1922	[47]		yes	Tropical Africa, Madagascar, Mayotte, Anjouan
		(*Eumelanomyia*)	*wigglesworthi*	Edwards, 1941	[47]		yes	Tropical Africa, Madagascar
		(*Oculeomyia*)	*bitaeniorhynchus*	Giles, 1901	This article		yes	Tropical Africa and Asia, China, Australia, Madagascar
		subgenus uncertain	sp. A		This article		yes	Mayotte
	*Eretmapodites*		*quinquevittatus*	Theobald, 1901	[96] as *Er. condei*		yes	Tropical Africa, Madagascar, Comoros Archipelago
			*subsimplicipes*	Edwards, 1914	[97]		yes	East and South Africa, Madagascar, Comoros Archipelago
	*Fredwardsius*		*vittatus*	(Bigot, 1861)	[70]		yes	Tropical Africa, Madagascar, Mayotte, Anjouan, Grande Comore
	*Lutzia*	(*Metalutzia*)	*tigripes*	(de Grandpre & de Charmoy, 1901)	[70]		yes	Tropical Africa, Madagascar, Mauritius, La Réunion, Comoros Archipelago
	*Mansonia*	(*Mansonioides*)	*uniformis*	(Theobald, 1901)	[47]		no	Oceania, Asia, Africa, Madagascar, Mayotte
	*Mimomyia*	(*Ingramia*)	*grjebinei*	(Brunhes, 1977)	[47]		yes	Endemic to Mayotte, Mohéli
		(*Ingramia*)	*roubaudi* *	(Doucet, 1950)	[94] as *Ravenalites roubaudi*		-	Endemic to Madagascar [19]
	*Neomelaniconion*		*circumluteolus*	(Theobald, 1908)	[47]		yes	Tropical Africa, Madagascar, Mayotte
	*Orthopodomyia*		*comorensis*	Brunhes, 1977	[47]		yes	Endemic to Mayotte
			*joyoni*	Brunhes, 1977	[47]		yes	Endemic to Grande Comore, Mohéli [52]
	*Polyleptomyia*		*albocephala*	(Theobald, 1903)	This article	[98]	yes	Tropical Africa, Madagascar, Seychelles, Mayotte, Mohéli
	*Skusea*		*cartroni*	Ventrillon, 1906	[99]		yes	Western Madagascar, Mayotte, Anjouan, Mohéli
			*pembaensis* *	Theobald, 1901	[100]		-	East African coast [48]
	*Stegomyia*		*albopicta*	(Skuse, 1895)	[101]		yes	From Japan to India, Madagascar, La Réunion, Mauritius, Seychelles; now invasive on all continents with the exception of Antarctic
		(*Mukwaya*)	*bromeliae*	Theobald, 1911	[94] as *Ae. simpsoni*	[94] as *Ae. simpsoni*; [49]	yes	Tropical Africa
		(*Mukwaya*)	*lilii* *	Theobald, 1910	[34]	[49]	-	Tropical Africa [57]
		(*Stegomyia*)	*aegypti*	(L., 1762)	[100]	[68]	yes	Pantropical
		(*Stegomyia*)	*pia*	Le Goff & Robert, 2013	[57]		yes	Endemic to Mayotte
	*Uranotaenia*	(*Pseudoficalbia*)	*comorensis*	da Cunha Ramos & Brunhes, 2004	[53]		yes	Endemic to Grande Comore, Mayotte
		(*Pseudoficalbia*)	*douceti* *	Grjebine, 1953	[47]		-	Endemic to Madagascar [53]
		(*Pseudoficalbia*)	*laffosseae*	da Cunha Ramos & Brunhes, 2004	This article		yes	Madagascar, Mayotte
		(*Pseudoficalbia*)	*pandani* *	(Theobald, 1912)	[47]		-	Endemic to the granitic Seychelles [53]
		(*Uranotaenia*)	*alboabdominalis*	Theobald, 1910	[47]		no	Tropical Africa, Mayotte, Madagascar
		(*Uranotaenia*)	*andavakae*	Doucet, 1950	[47]		yes	Madagascar, Mayotte
		(*Uranotaenia*)	*anopheloides*	Brunhes & Razafindrasolo, 1975	[53]		no	Madagascar, Mayotte
		(*Uranotaenia*)	*mayottensis*	Brunhes, 1977	[47]		yes	Madagascar, Mayotte
	*Zavortinkius*		*brunhesi*	Reinert, 1999	[102]		perhaps (see text)	Endemic to Mayotte
			*monetus*	(Edwards, 1935)	[94]		perhaps (see text)	Madagascar, Mayotte, Mohéli, Glorioso, Juan De Nova

The five species marked with an asterisk are considered herein as not occurring definitively on the island and not included in our tabulations.

* Species not occurring definitively on Mayotte.

Within our 2008–2012 collections, seven species were documented on Mayotte for the first time (*Anopheles merus*, *Culex bitaeniorhynchus*, *Cx. tritaeniorhynchus*, an undetermined *Culex* sp. A, *Polyleptiomyia albocephala*, *Stegomyia pia* and *Uranotaenia laffosseae*). In addition, *Orthopodomyia joyoni* was reinstated to the Mayotte list. In total, eight taxa were integrated for the first time or reinstated to the known fauna of Mayotte. With the exceptions of *Culex* sp. A, the identity of which is uncertain (see below “Difficulties with identifications at the species level” and text A in File S1), and *St. pia*, described as new to science based on our collections [57], all of the six remaining species (*An. merus*, *Cx. bitaeniorhynchus*, *Cx. tritaeniorhynchus*, *Po. albocephala*, *Ur. laffosseae* and *Or. joyoni*) were previously known from Madagascar and/or other islands in the Comoros Archipelago (Table 3).

As one of our intentions associated with the different inventoried habitats (see below for further details), was to estimate relative abundance of the locally occurring mosquito taxa, we employed the following classification.

#### Principal taxa

Species collected in ≥20% of the inventories habitats are considered as ‘principal species’ and 27 taxa fell into this category (Table 3) and the most common were *St. aegypti* (70 habitats), *St. albopicta* (57), *Anopheles gambiae* (67), and *Eretmapodites subsimplicipes* (61); these taxa represent 35% of the species recorded and occurred in 52% of sampled habitats. The first three species are well known vectors of viruses and parasites. If our sampling efforts had included more urban areas, *Cx. quinquefasciatus* would have been probably better represented in our collections [65]; this species is the principal vector of Bancroft filaria on Mayotte [21], [66].

#### Less common taxa

Species collected at an incident rate of less than 20% at a given habitat are considered ‘rare’ and 14 taxa fell into this category (Table B in File S1), which represents 6% of species recorded and occurred in 11% of sampled habitats. These less common taxa were principally from habitats other than human made containers and for which larval sampling can be inefficient. Given the abundance of such containers on Mayotte, non-container breeding mosquito species are likely to be under represented in our sampling.

### Difficulties with identifications at the species level

In the majority of cases, it was possible to identify specimens with considerable confidence to the species level using morphological criteria. However, a few cases posed some problems: genus *Orthopodomyia*, subgenus *Mukwaya* of the genus *Stegomyia*, *Zavortinkius monetus* group, *Culex decens* subgroup and *Cx.* sp. A. These aspects are discussed in Text A in File S1.

When morphological characters for species determination did not produce conclusive results, we employed molecular techniques. The usefulness of this approach is demonstrated by the *Anopheles gambiae s.s.* complex. Amongst the 79 specimens collected on Mayotte and referable to this complex, all were determined as *An. gambiae s.s.*, with one exception, which was collected on 23 March 2011 in rain water puddle – turbid water – on Petite-Terre (MY 87bis), and associated with six larvae of *An. gambiae s.s.* The habitat was about 100 m from the coast. *Anopheles gambiae s.l.* was probably introduced to Grande Comore around 1920, but its establishment on the three other islands in the archipelago dates from earlier periods [19], [67]. The occurrence of *An. merus* in the archipelago was not accepted by certain workers [68] or other researchers, based on morphological criteria (palpal ratio, antennal coeloconic sensilla, branching of the seta 1-P) reported it on Grande Comore [46], [69], [70] and with a preference for habitats with high salt concentrations. However, these criteria are not definitive and when using different techniques (cytogenetics and molecular genetics), *An. gambiae s.s.* was identified on different islands in the archipelago [71]–[74]. To our knowledge, we present herein the first indisputable evidence of *An. merus* on Mayotte, which is concordant with recent data that this species represents 2% of the *An. gambiae* complex on Grande Comore, Anjouan and Mohéli [20]. These findings are not surprising, as *An. merus* occurs both on continental Africa and Madagascar [51], [75].

### The effectiveness of surveys and measures of species richness

**Figure 2:**
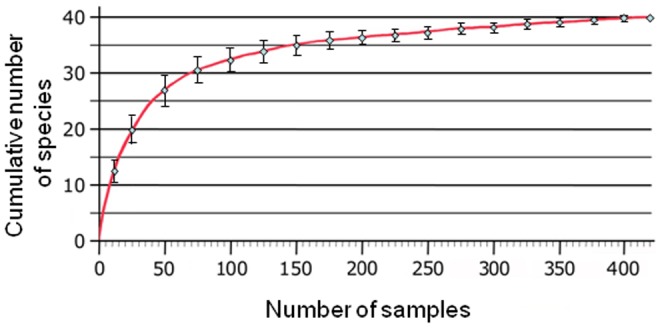
Species accumulation curve for the 40 mosquito species observed on Mayotte at 420 habitats during the 2008–2012 surveys. Bars indicate two standard deviations.

In total, 40 species were documented during our surveys at 420 habitats with at least one larva or pupa. The species accumulation curve is presented Figure 2, which approaches an apparent asymptote. The 20^th^, 30^th^, 39^th^ and 40^th^ species were observed in the 25^th^, 69^th^, 314^th^ and 412^th^ habitats, respectively. The first order jack-knife estimation of total species richness based on successively large number of samples from the data set indicates that the survey was notably comprehensive. By extrapolation, considerable effort would need to be expended to add other potentially rare species to the Mayotte Culicidae list.

### Mosquito species richness in relation to types of larval habitats

**Figure 3:**
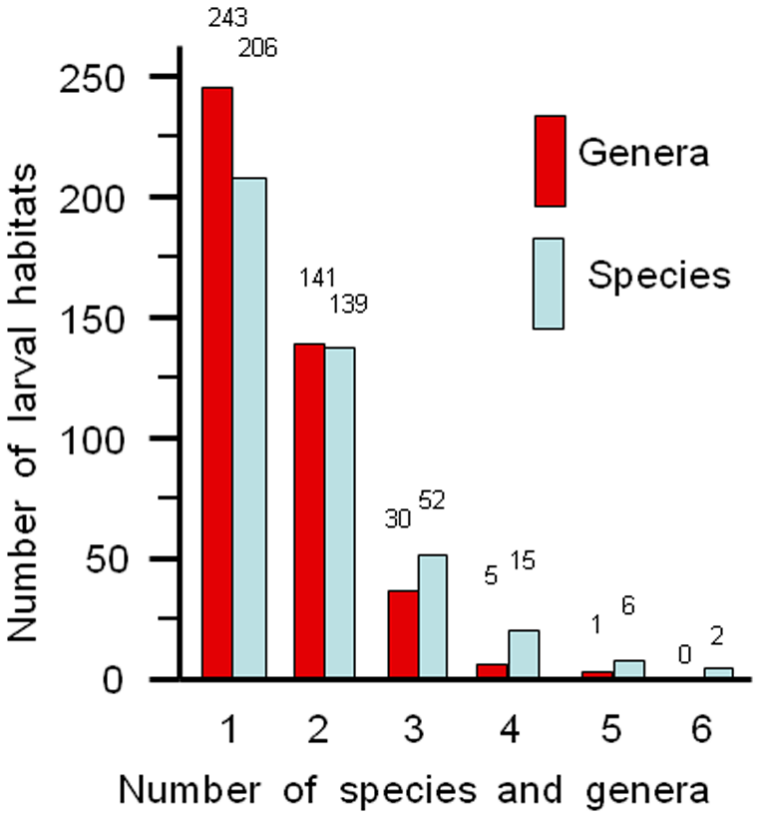
Frequency histograms for the number of mosquito genera and species versus the number of larval habitats (n = 420) on Mayotte during the 2008–2012 surveys.

Across Mayotte, 48% of the 420 habitats were occupied by a single mosquito species and hence, the co-occurrence of multiple taxa was the majority rule. The maximum number of species and genera at a single habitat was six and five, respectively (Fig. 3). Mean species richness in the different habitats ranged from 1.00 to 3.29 and this was associated with habitat type. For example, snail shells (introduced *Achatina*), axils of banana leaves and tree-holes were the habitats with the lower richness and axils of *Typhonodorum*, abandoned tires and marsh and swamp water were the habitats with the higher richness (Table 4). *Stegomyia albopicta* was preferentially found in disposed solid waste, *St. aegypti* in coconut husks and cut bamboo, *St. bromeliae* in axils of banana and *Typhonodorum*, *Zavortinkius monetus* gr. and *Orthopodomyia comorensis* in tree-holes, *Skusea cartroni* in crab-holes, *Eretmapodites subsimplicipes* in coconut husks and fallen leaf litter, *Uranotaenia comorensis* on mushroom caps, and *Culex simpsoni*, *Anopheles gambiae* and *An. coustani* in marsh and swamp water.

**Table 4:** Occurrence on Mayotte during the 2008–2012 surveys of the 27 principal mosquito species when present in more than 20% of at least one type of larval habitat.

		*Aedimorphus*	*Anopheles*		*Culex*									*Eretmapodites*		*Fredwardsius*	*Lutzia tigripes*	*Mimomyia grjebinei*	*Orthopodomyia comorensis*	*Stegomyia*				*Skusea cartroni*	*Uranotaenia*			*Zavortinkius monetus gr.*	TOTAL
	n	*fowleri*	*coustani*	*gambiae*	*antennatus*	*carleti*	*decens*	*horridus*	*nebulosus*	*quinquefasciatus*	*simpsoni*	*tritaeniorhynchus*	*wigglesworthi*	*quinquevittatus*	*subsimplicipes*	*vittatus*	*tigripes*	*grjebinei*	*comorensis*	*aegypti*	*albopictus*	*bromeliae*	*pia*	*cartroni*	*comorensis*	*laffosseae*	*moyottensis*	*monetus gr.*	
Snail shell	1													**1**															1
Axils of banana/pineapple leaves	14																	2		1		**12**				1			16
Tree-hole	70	1		1				8	2						2	1	1		**24**	9	6		8	1				**28**	92
Crab-hole	7			1	1						1													**6**					9
Holes in rock	27			1			2			1	1		3		1	**5**			1	**7**	2	1	**5**		2		**1**	2	35
Slow flowing water	22		**11**	**6**	1										1												**3**		22
Fallen leaf litter	26														**16**					**6**	2				**14**				38
Disposed solid waste	47			1		1		2	1	3				4	**10**		2		2	**16**	**24**	3	1	1	5				76
Coconut husk	16							1	**3**						**11**					**6**	1	1	2					2	27
Axils of taro/*Typhonodorum* plants	21														1			**14**	0	2	1	**21**				**5**			44
Large artificial containers	14			2			2			**3**	1					1	**4**			**5**	**3**				2				23
Holes in ground	8	**2**	**2**	1	1			1					**2**				1				**2**			**2**					14
Mushroom caps	4														**3**										**3**				6
Water ponds and pools	67	**13**	7	**42**	6		8			3	**15**	1	7				9				2				1		1	1	116
Cut bamboo	45					**9**		**9**	7						**12**				**10**	**13**	**9**	1	**11**		5			4	90
Vegetable and plant matter	9						1		1						**4**	1			1	**3**	1	1	1		**4**			1	19
Discarded tires	5			1			**2**			1			1				**2**			**2**	**3**	1							13
Marsh and swamp water	17	1	**11**	**11**	**6**		3				**8**	**4**	2				2				1			1					50
TOTAL	420	17	31	67	15	10	18	21	14	11	26	5	15	5	61	8	21	16	38	70	57	41	28	11	36	6	5	38	691

Sites are listed by increasing mosquito species richness and the species in alphabetic order. (Values in bold derived from Table 2.)

**Figure 4:**
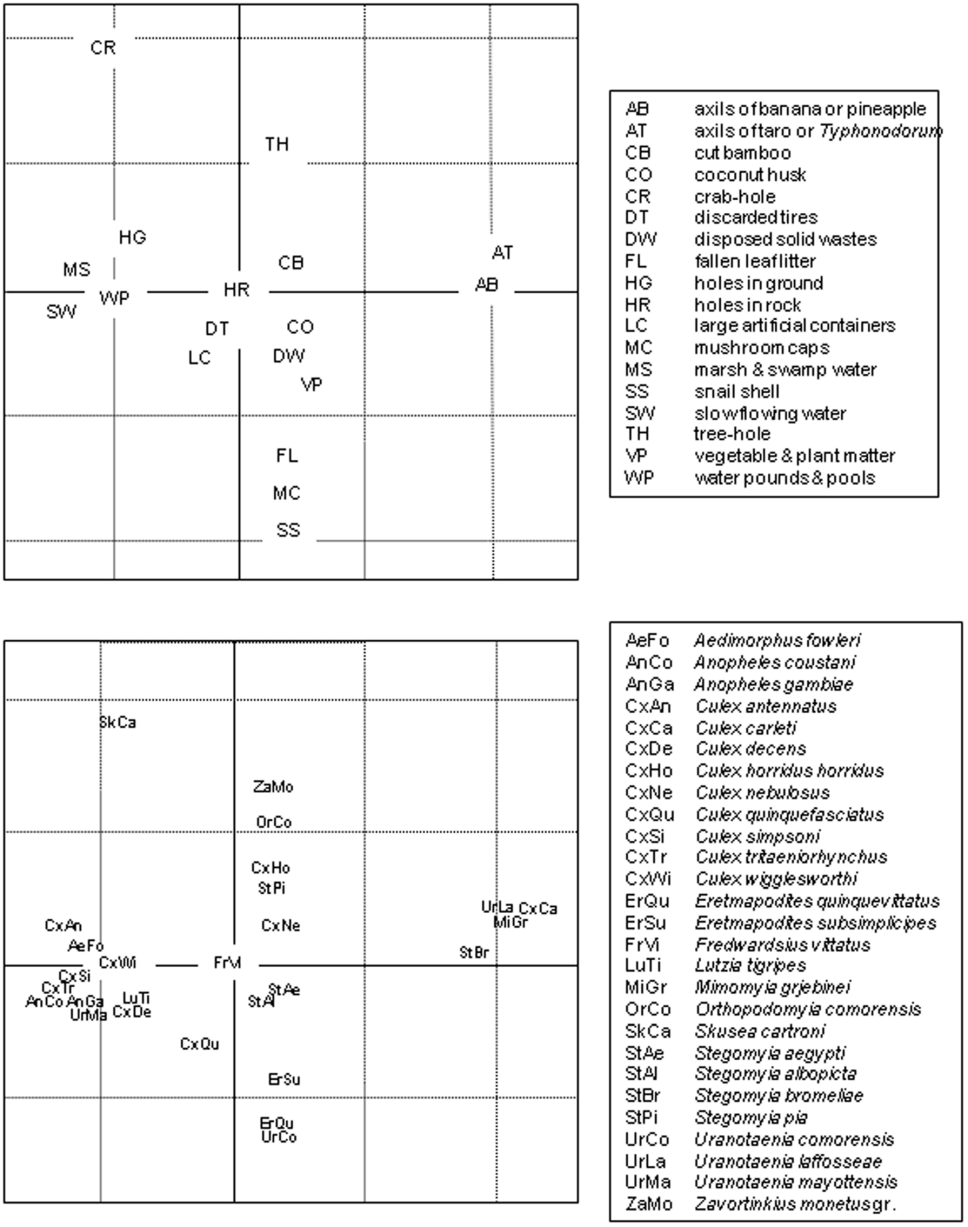
Correspondence analysis for the 18 main types of larval habitats *vs.* the 27 principally collected mosquito species. Represented here are axis 1 (horizontal) and axis 3 (vertical) that explained 33.3% (22.9%+11.4%) of total variance; the scales are equal for the two graphs (grid step size = 1). The separation along axis 1 follows a temporal gradient from non-permanent (axils of banana-pineapple, taro and *Typhonodorum* plants) to permanent habitats (marsh and swamp water, water pools during the rainy season and slow flowing water). Axis 3 displays another gradient from habitats with restricted openings (crab-holes, tree-holes) to habitats with open access (mushroom caps, fallen leaf litter and snail shells).

In the correspondence analysis focusing on 18 different habitat types and 27 mosquito species, the first five axes explained 22.9%, 21.0%, 11.4%, 10.5% and 7.6% of the total variance. A two-dimensional graph plotting axes 1 and 2 (Fig. A in File S1) presented two different clusters of habitat types – the first includes axils of banana-pineapple plants and axils of taro-*Typhonodorum*, and the second cluster includes the 16 other habitats. In Figure 4, we present graphs of axes 1 and 3 and the associated interpretation is given in the figure caption. In several cases, certain mosquito species are restricted to specific habitat types; for example, *Sk. cartroni* in crab-holes, *Mimomyia grjebinei* in the axils of taro and *Typhonodorum*, *Er. quinquevittatus* in snail shells and *Cx. simpsoni* occurring in marsh and swamp water and water pools.

### Relationships between mosquito species

According to Merritt and colleagues [76], mosquito larvae can be classified according to their functional role within an ecosystem: 1) ‘filterers’ collect suspended food particles at the air-water interface (most *Anopheles* and *Uranotaenia* of the subgenus *Uranotaenia*) and in the water column (many *Culex*, some Aedini); 2) ‘gatherers’ collect particles settled on the substratum (many Aedini); 3) ‘scrapers’ remove food adhering to various substrata (*Uranotaenia* of the subgenus *Pseudoficalbia*); 4) ‘shredders’ feed on plants or dead organisms (some *Culex*, some Aedini); and 5) ‘predators’ attack living animal prey (*Lutzia tigripes*). Most culicine species employ at least two feeding modes although one of these is predominantly employed [77]. This variety of feeding modes, most probably, allows the co-occurrence of several mosquito species in the same specific habitat.

In order to document mosquito species associations, tests were performed using the Fisher's exact test of independence for each species pair and each habitat type. Among the 16 mosquito species pairs examined, no positive association (co-occurrence observed at frequency higher than expected by chance) was found (Table C in File S1). However, exclusion of species (co-occurrence observed at frequency lower than expected by chance) was observed in two cases.

#### *Zavortinkius monetus* group – *Orthopodomyia comorensis* in tree-holes

In the 70 tree-hole habitats examined, 20 were lacking these two taxa, 26 only had individuals of the *Za. monetus* group, 22 only with *Or. comorensis*, and two with both taxa (P = 0.0001, Fisher's exact test). There appears to be elevational segregation between these taxa, with members of the *Za. monetus* group and *Or. comorensis* occurring at mean elevations of 58 m and 200 m, respectively (P = 0.001, Mann-Whitney *U* test). Further, some ecological preferences were noted, such as water colour, probably associated with tannin concentration. Members of the *Za. monetus* group were found in clear or slightly coloured water and *Or. comorensis* in brown to black water (P = 0.069, Fisher's exact two-tailed test, and not significant). Hence, there is little evidence of competitive exclusion between these two species and the observed separation is probably related to aspects of their ecology, specifically elevational range.

#### *Stegomyia albopicta* – *Eretmapodites subsimplicipes* in abandoned solid waste

In the 47 habitats falling under this habitat type, 13 habitats did not contain these two species, 24 only had *St. albopicta*, 10 only had *Er. subsimplicipes*, and in no case were the two found together (P = 0.0002, Fisher's exact test). No clear explanation can be found for this observation based on aspects of the natural history of these species (e.g., elevation or water colour). A possible suggestion may be predation by *Er. subsimplicipes* [78]. An alternative suggestion, although not demonstrated here due to limited samples, is a preference of *St. albopicta* for abandoned solid waste habitats (plastic associated with discarded cars in zones with anthropogenic degradation or urban areas) and *Er. subsimplicipes* in rural and wooded areas. Other examples of the co-occurrence of these two species in different habitat types do not suggest competition between them. They were found together in two leaf litter habitats and in two cut bamboo habitats.

### Biodiversity and biogeography of mosquitoes on Mayotte

In total, 50 species of mosquitoes have been cited in the literature to occur on Mayotte. However, in five cases based on reference specimens, some problem exists with the species identifications and these taxa are not accepted to the island's mosquito fauna (see Table 3 for this list, associated references, and more recent identification updates). Adding our results to previous work, the mosquitoes documented on Mayotte currently includes 45 species belonging to 15 genera.

**Figure 5:**
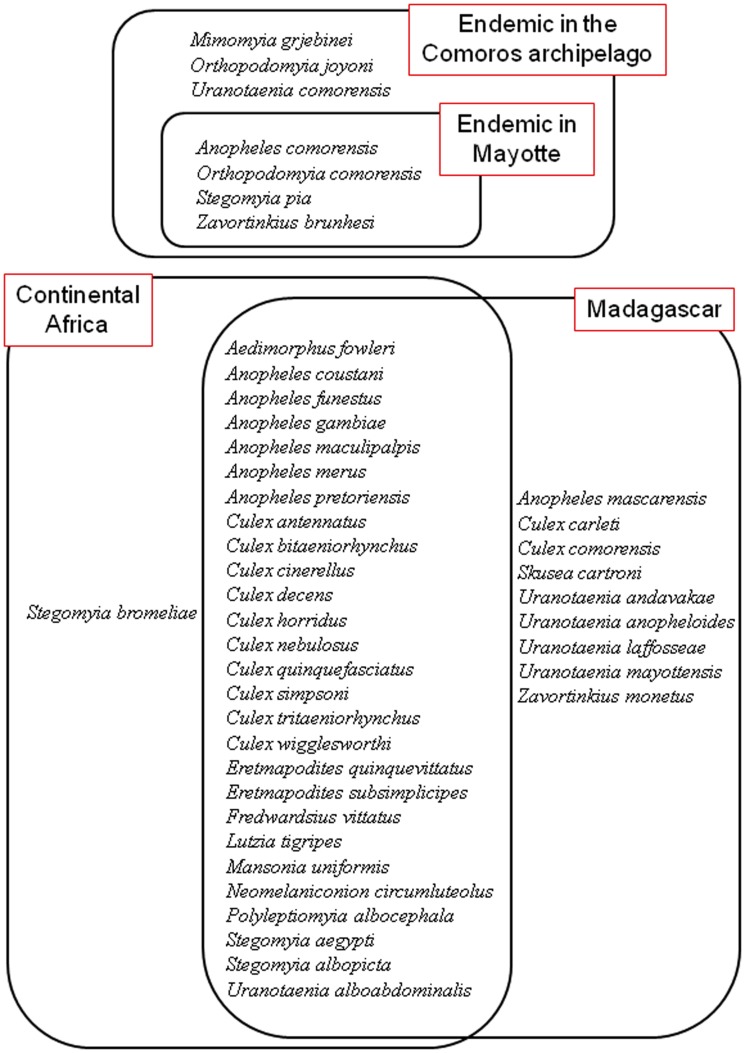
Diagrammatic representation of the known distribution of the 44 mosquito species occurring on Mayotte. Four species are considered as endemic to Mayotte and three others endemic to the greater Comoros Archipelago. Most species occur on Madagascar and the African continent, and of biogeographic interest, 10 are shared with Madagascar and one with the African continent. *Culex* sp. A is not listed as its specific identification needs further documentation (see Text S1). Species are listed in alphabetic order.

Amongst these 45 species, four species are considered endemic to Mayotte and three others endemic to the Comoros Archipelago. Amongst the remaining 37 taxa, a large portion include species also found on Madagascar and the African continent (Fig. 5), and by consequence, these taxa provide little insight into their biogeographic origins. Given the relatively recent *in situ* origin of Mayotte [79] and the nearly equidistant position of the island between Africa and Madagascar, at least based on chance, the landmass of origin of mosquitoes is in principal equally likely between these two source areas. However, of the more geographically restricted taxa occurring on Mayotte, 10 are shared with Madagascar and one with the African continent. It is of interest to note that this biased dispersal from east to west follows dominant wind direction [80] and not that of human trade activities. Based on the Regional Customs Department on Mayotte, importation of commercial goods to the island from Africa as compared toMadagascar was 25 times higher in tonnage and 10 times higher in value [81].

Such a disproportionate percentage of mosquito species of Malagasy origin on islands in the Comoros is found in other organisms, for example insects [82], bats [83] and native terrestrial birds [84]. Further, a number of groups, genera or species, such as baobabs, ants, caddisflies, scorpions and chameleons, thought to be restricted to Madagascar, have been recently found on Mayotte [85]–[90], underlining the close biogeographic relations of Madagascar as a source area for colonization of Mayotte.

The diversity of 45 species and 15 genera of mosquitoes on Mayotte is considerable, particularly given the island's modest elevation range and relatively small surface area. To put these figures into perspective concerning other islands in the southwestern Indian Ocean, the Culicidae of the Seychelles encompasses 21 species in eight genera [54], [91], La Réunion 12 species in seven genera [55], Grande Comore 12 species in seven genera, Mauritius 17 species in six genera, and Rodriguez three species in two genera [92]. These figures are not available for the other islands in the Comoros Archipelago (Anjouan and Mohéli), but a few aspects can be highlighted to explain the relative richness of Culicidae on Mayotte. It is the oldest island in the Comoros Archipelago, having formed *in situ* some 15 million years ago, while the youngest island in the chain is Grande Comore, being less than 0.5 million years old [79]. Hence, Mayotte has had more time to accumulate species through over water colonization. An interesting comparison to the mosquitoes of the Comoros Archipelago, is that of the Mascarene Archipelago (La Réunion, Mauritius and Rodriguez), which is located about 700 km to the west of Madagascar and several thousand kilometres to the nearest portion of Asia. The Mascarene Archipelago formed *in situ* about 5 million years ago [93], and while it has a greater elevational range than Mayotte, its level of isolation combined with being positioned in the opposite direction of the easterlies from Madagascar, has resulted in a distinctly more species depauperate mosquito fauna than the Comoros Archipelago. Of particular interest concerning Mayotte, the relative richness of mosquito taxa appears to remain stable, even in light of human population increase and associated degradation of natural environments.

## Conclusions

On the basis of an intensive survey, following the style of “All-Taxa Biological Inventory”, of Culicidae mosquito pupae and larvae at 420 habitats on Mayotte in the Comoros Archipelago, which resulted in about 6,000 collected specimens, combined with previous mosquito inventories conducted on the island, 45 species belonging to 15 genera have been documented on Mayotte. With the use of classical morphological characters, as well as molecular genetic markers, seven taxa were recorded on the island for the first time, with at least one of these being new to science; in addition, one species was reinstated to the island's mosquito list. Among these height taxa, there is one potential vector of human malaria (*An. merus*) and another potential vector of human arboviruses (*St. pia*).

This work highlights the importance of detailed analysis of different mosquito larvae and pupae occurring at different localities and in different habitat types for detailed insight into the island wide fauna. The species accumulation curve indicates that the survey approached being exhaustive. Hence, by extrapolation, it is possible in a restricted geographical zone such as Mayotte (374 km^2^) to produce a nearly complete faunal list of an insect group such as mosquitoes through concentrated efforts.

Information on the different species occurring at inventoried aquatic habitats provides interesting ecological insights into species co-occurrence and habitat preferences. These data suggest that on Mayotte and probably on other tropical islands with relatively restricted size and ecological conditions, Culicidae mosquitoes can be used as biological indicators associated with shifting ecological conditions and changes in species diversity and richness. These aspects have critical implications for measures of the impact of invasive introduced mosquitoes, particularly those implicated in the transmission of human pathogens, as well as aspects such as climatic change. Further, given the natural habitat specificity of certain mosquito taxa, they represent a group that needs to be considered in current conservation efforts of remaining natural forested habitats on the island.

## Supporting Information

File S1 **Supporting Information.** Text A, Entomological difficulties to identify specimens to species level. Table A, Distribution of the 420 types of larval habitat during the 2008–2012 surveys. Table B, Occurrence of the 14 rare mosquito species collected on Mayotte during the 2008–2012 surveys. Table C, Statistical analysis for each species pair and type of larval habitat. Figure A, Correspondence analysis for the 18 main types of larval habitats *vs.* the 27 principally collected mosquito species.
(PDF)
