# Supplementary material for: Survey of the Mosquitoes (Diptera: Culicidae) of Mayotte
Source: PLoS One. 2014 Jul 8;9(7):e100696. doi: 10.1371/journal.pone.0100696 (PMC4086827; doi:10.1371/journal.pone.0100696)
Supplement: File S1 — Supporting Information. Text A, Entomological difficulties to identify specimens to species level. Table A, Distribution of the 420 types of larval habitat during the 2008–2012 surveys. Table B, Occurrence of the 14 rare mosquito species collected on Mayotte during the 2008–2012 surveys. Table C, Statistical analysis for each species pair and type of larval habitat. Figure A, Correspondence analysis for the 18 main types of larval habitats vs. the 27 principally collected mosquito species. (PDF) [file pone.0100696.s001.pdf]

Supporting information

Le Goff G, Goodman SM, Elguero E & Robert V (2014) Survey of the mosquitoes (Diptera: Culicidae) of Mayotte. PLoS One.

Text A. Entomological difficulties to identify specimens to species level.

***Orthopodomyia comorensis* vs. *Or. joyoni*.** These two closely related species were initially recorded as endemic to Mayotte (*Or. comorensis*) or endemic to Grande Comore, Mayotte and Mohéli (*Or. joyoni*) [47]. Subsequently, Mayotte was not considered part of the range of *Or. joyoni* [52]. The types of these two species have been examined (deposited in the ARIM collection) and based on morphology of larval *Orthopodomyia*, the following points can be presented. The most useful character to separate these two species is the morphology of seta 1-III, which is a single and very long structure in *Or. joyoni* or of more moderate length and 3 branched structure in *Or. comorensis*; these differences also separate the two species at larval stage 3 and 4. Adults are easier to distinguish based on abdominal terga ornamentation, although the captions in Figures 9 and 10 in the original description [47] have been reversed. *Orthopodomyia comorensis* is the dominant species on Mayotte (38 habitats) and *Or. joyoni* is less frequent (5 habitats); at one habitat the two species occurred in sympatry.

***Stegomyia bromeliae* vs. *St. lilii*.** The distinction between these two closely related-species, which belong to the *St. simpsoni* complex, was recently established, and all of our material is referable to *St. bromeliae* (see [57]). These conclusions are in agreement with a recent study conducted on Grande Comore, Anjouan and Mohéli [20].

***Zavortinkius monetus* group, including *Za. monetus* vs. *Za. brunhesi*.** The original description of *Za. monetus* was based on two females from Madagascar [43]. Subsequently, larvae, pupae and males of this species were diagnosed [47], which occurs in western Madagascar [Ravahonjanahary C (1978) Les *Aedes* de Madagascar (Diptera-Culicidae). 1- Etude monographique du genre. 2- Biologie d'*Aedes* (*Diceromyia*) *tiptoni*. Paris: Travaux et Documents de l'ORSTOM. 210 p.], Mayotte and Mohéli [47]. The description of *Za. brunhesi* was based on two males from Mayotte [102], and without associated material of larvae, pupae and females. The two male types (ARIM E387) from near Dembeni, Mayotte, were identified from a preimaginal collection obtained from a hole in a bamboo, which also included larvae, pupae and females. The absence of white scales on the postspiracular area tends to be constant for both males and females, although there are a few exceptions. However, aspects of the thoracic scales thought to be diagnostic of *Za. brunhesi* (ornamentation of mesopostnotum and posterior scutum) appeared to be inconsistent and, in our opinion, not useful to distinguish it from *Za. monetus*. We examined three male genitalia (without dissection) of *Za. brunhesi*, which were similar in morphology to those of *Za. monetus* (without dissection [47]). Further, the dissected genitalia of an individual of *Za. monetus* was similar to the dissected genitalia of *Za. brunhesi* [102]. Using morphological criteria, we were unable to assign definitively a species name to our Mayotte specimens, which show similarities to *Za. monetus* and *Za. brunhesi*, and this material was assigned to the *Za. monetus* group. Probably only one of these two species occurs on Mayotte, but further morphological and molecular studies are needed to properly diagnose these taxa, including material from Madagascar and other islands in the Comoros.

***Culex decens* vs. *Cx. decens* group.** The subgroup *Cx. decens* (herein *Cx. decens* gr.) belongs to the *Pipiens* group of the *Culex* subgenus. *Culex decens* gr. includes 8 Afrotropical Region species [Harbach 1988] of which 3 have been recorded in the Malagasy subregion (*Cx. antennatus*, *Cx. decens* and *Cx. perfidiosus*). The morphology of females of these species is relatively similar but male genitalia of *Cx. perfidiosus* are distinctive, especially on the style. At the larval stage, *Cx. antennatus* and *Cx. decens* are very similar, but Harbach stated that the setae 1-IV and V permit the distinction between the two species, despite of their broad geographical distribution [Harbach RE (1988) The mosquitoes of the subgenus *Culex* in Southwestern Asia and Egypt (Diptera: Culicidae). Contrib Amer Ent Ins 24(1): 240 p.]. The larvae of *Cx. inviduosus* (an Afrotropical species also belonging to the *Cx. decens* gr.) is thought to be indistinguishable from *Cx. decens* (see [44]), but *Cx. inviduosus* is unknown in southern Africa and Malagasy subregion. During the 2008-2012 survey, using Harbach's criteria, we identified *Cx. decens* based on 16 larvae and 2 adults (1 male and 1 female emerged from pupae collected at MY 23). Finally, we are of the opinion, after Brunhes [47] on Mayotte and in agreement with Marsden *et al.* [20] on Grande Comore, Anjouan and Mohéli, that this species is the only taxon of the *Cx. decens* group occurring in the Comoros Archipelago.

***Culex* species A.** This taxon is only known during the 2008-2012 surveys from three specimens, all of which are 2nd instars, collected in pool (MY 637, two larvae) and cut bamboo (MY 168, one larva). These larvae show five distinctive criteria: (i) setae 5-C and 6-C single and aciculate, (ii) saddle clearly incomplete ventrally, (iii) caudal setae (2-X and 3-X) simple, (iv) ventral brush (4-X) with 4 pairs of shorter bristles, and (v) siphon sub-cylindrical with its basal part only 1/3 larger than the terminal one, siphonal index 6 or 7, distinctly sclerotised, seta 1-S composed of 4 or 5 small tuffs, each tuff 2 short branches with length lower than the siphon diameter; pecten with 8-9 sharp teeth, every tooth has 7-9 sharpened denticules on the internal edge in the form of a half-harpoon. This undetermined *Culex* sp., here provisionally referenced *Culex* sp. A, is new for Mayotte.

In our Collection d'Arthropodes d'Intérêt Médical (ARIM), Laboratoire de Taxonomie des Vecteurs, Institut de Recherche pour le Développement, Montpellier, France, we found a larva collected on Mayotte, Mamoutzou, 11 February 1956, Grjebine rec,, LAM412. We believe that this larva, probably a 3rd instars or less probably a 4th instars, belongs to *Culex* sp. A. It is presented below (abdomen, distal part, lateral view; head, dorsal view).

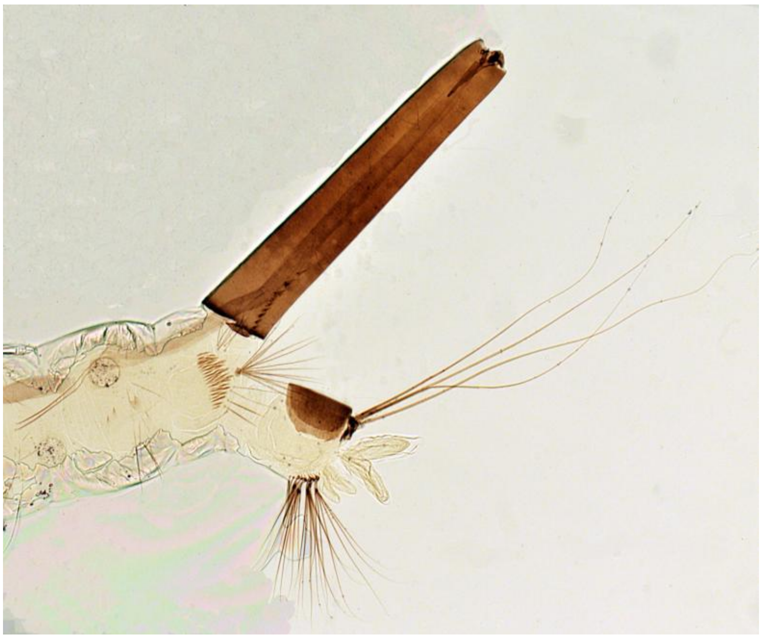

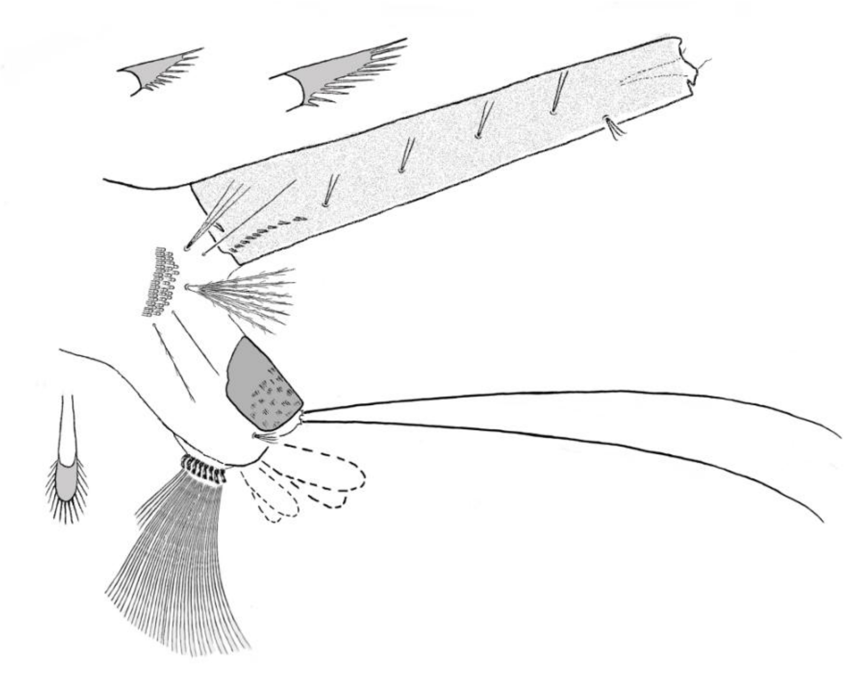

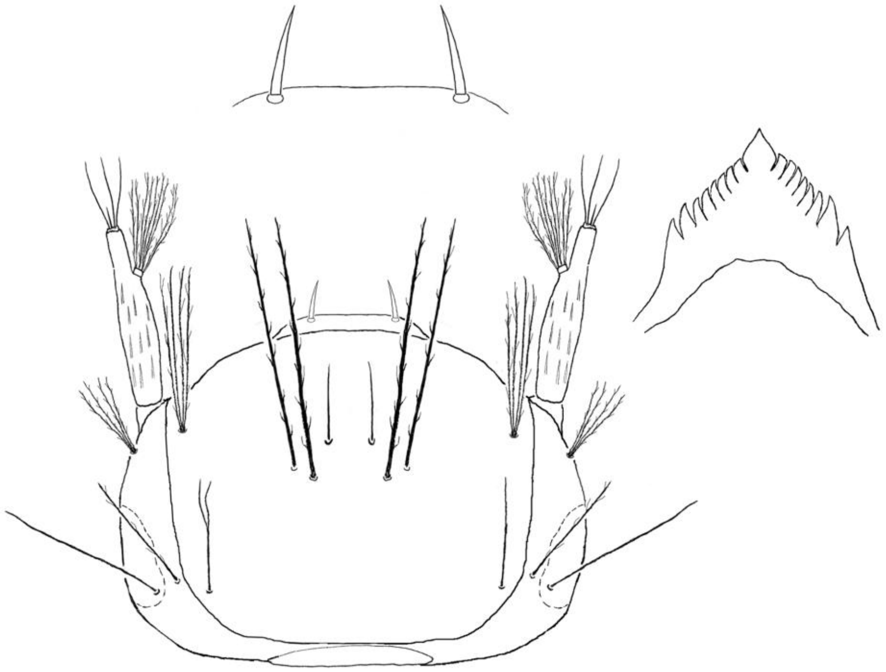

Table A. Distribution of the 420 types of larval habitat during the 2008-2012 surveys.

Main type of habitat	n (%)	Type of habitat	n (%)
Natural containers of vegetal origin	205 (48.8)	Axils of banana leaves	7 (1.7)
		Axils of pineapple plants	7 (1.7)
		Axils of taro plants	4 (0.9)
		Axils of *Typhonodorum* plants	17 (4.0)
		Tree-holes	70 (16.7)
		Cut bamboo	45 (10.7)
		Fallen leaf litter	26 (6.2)
		Coconut husk	16 (3.8)
		Mushroom caps	4 (1.0)
		Vegetable and plant matter	9 (2.1)
Natural containers of animal origin	1 (0.3)	Snail shells	1 (0.3)
Pools of water of different size and origin	148 (35.2)	Crab-holes	7 (1.7)
		Holes in ground	8 (1.9)
		Holes in rock	27 (6.4)
		Slow flowing water	22 (5.2)
		Water ponds and pools	67 (16.0)
		Marsh and swamp water	17 (4.0)
Artificial human-made containers	66 (15.7)	Disposed solid waste	47 (11.2)
		Discarded tires	5 (1.2)
		Large artificial container	14 (3.3)
Total	420 (100.0)		420 (100.0)

Table B. Occurrence of the 14 rare mosquito species (*i.e.* present in less than 20% of all types of larval habitats) collected on Mayotte during the 2008-2012 surveys. Habitats are listed by increasing species richness and the species in alphabetic order.

		Anopheles				*Culex*				*Neomelanoconion*	*Orthopodomyia*	*Polyleptiomyia*	*Uranotaenia*	*Zavortinkius*		TOTAL
	n	*funestus*	*mascarensis*	*merus*	*pretoriensis*	*bitaeniorhynchus*	*cinerellus*	*comorensis*	species A	*circumluteolus*	*joyoni*	*albocephala*	*andavakae*	*brunhesi*	*monetus s.s.*	
Snail shell	1															0
Axils of banana & pineapple leaves	14															0
Tree-hole	70						1	1			1			1	2	6
Crab-hole	7															0
Holes in rock	27				1						1					2
Slow flowing water	22	2			3	2		1					1			9
Fallen leaf litter	26						1									1
Disposed solid waste	47										1					1
Coconut husk	16															0
Axils of taro & *Typhonodorum* plants	21															0
Large artificial containers	14							1								1
Holes in ground	8							1								1
Mushroom caps	4															0
Water ponds and pools	67			1	3	1	2	8	1				1			17
Cut bamboo	45								1							1
Vegetable & plant matter	9						1				1					2
Discarded tires	5										1					1
March and swamp water	17		1							2		1	1			5
TOTAL	420	2	1	1	7	3	5	12	2	2	5	1	3	1	2	47

Table C. Statistical analysis for each species pair and type of larval habitat for those recorded in at least 10 habitats (16 species pair combinations and habitat type satisfied this criteria), using the Fisher's exact test of independence and the Bonferroni's correction for the alpha threshold (0.05/16 = 0.003). Habitats are listed by increasing species richness. S: significant; NS: not significant.

Type of habitat	Species pair	P	Bonferroni's correction
Tree-hole	*Zavortinkius monetus* group - *Orthopodomyia comorensis*	0.0001	S
Fallen leaf litter	*Eretmapodites subsimplicipes* - *Or. comorensis*	0.006	NS
Disposed solid waste	*Stegomyia aegypti* - *St. albopicta*	0.13	NS
Disposed solid waste	*St. aegypti* - *Er. subsimplicipes*	0.46	NS
Disposed solid waste	*St. albopicta* - *Er. subsimplicipes*	0.0002	S
Axils of taro & *Typhonodorum* plants	*St. bromeliae* - *Mimomyia grjebinei*	1	NS
Water pools	*Aedimorphus fowleri* - *Anopheles gambiae*	0.75	NS
Water ponds & pools	*Am. fowleri* - *Culex simpsoni*	0.47	NS
Water ponds & pools	*An. gambiae* - *Cx. simpsoni*	0.38	NS
Cut bamboo	*St. aegypti* - *St. pia*	1	NS
Cut bamboo	*St. aegypti* - *Er. subsimplicipes*	0.46	NS
Cut bamboo	*St. aegypti* - *Or. comorensis*	0.70	NS
Cut bamboo	*St. pia* - *Er. subsimplicipes*	0.45	NS
Cut bamboo	*St. pia* - *Or. comorensis*	0.41	NS
Cut bamboo	*Er. subsimplicipes* - *Or. comorensis*	0.25	NS
Marsh & swamp water	*An. coustani* - *An. gambiae*	1	NS

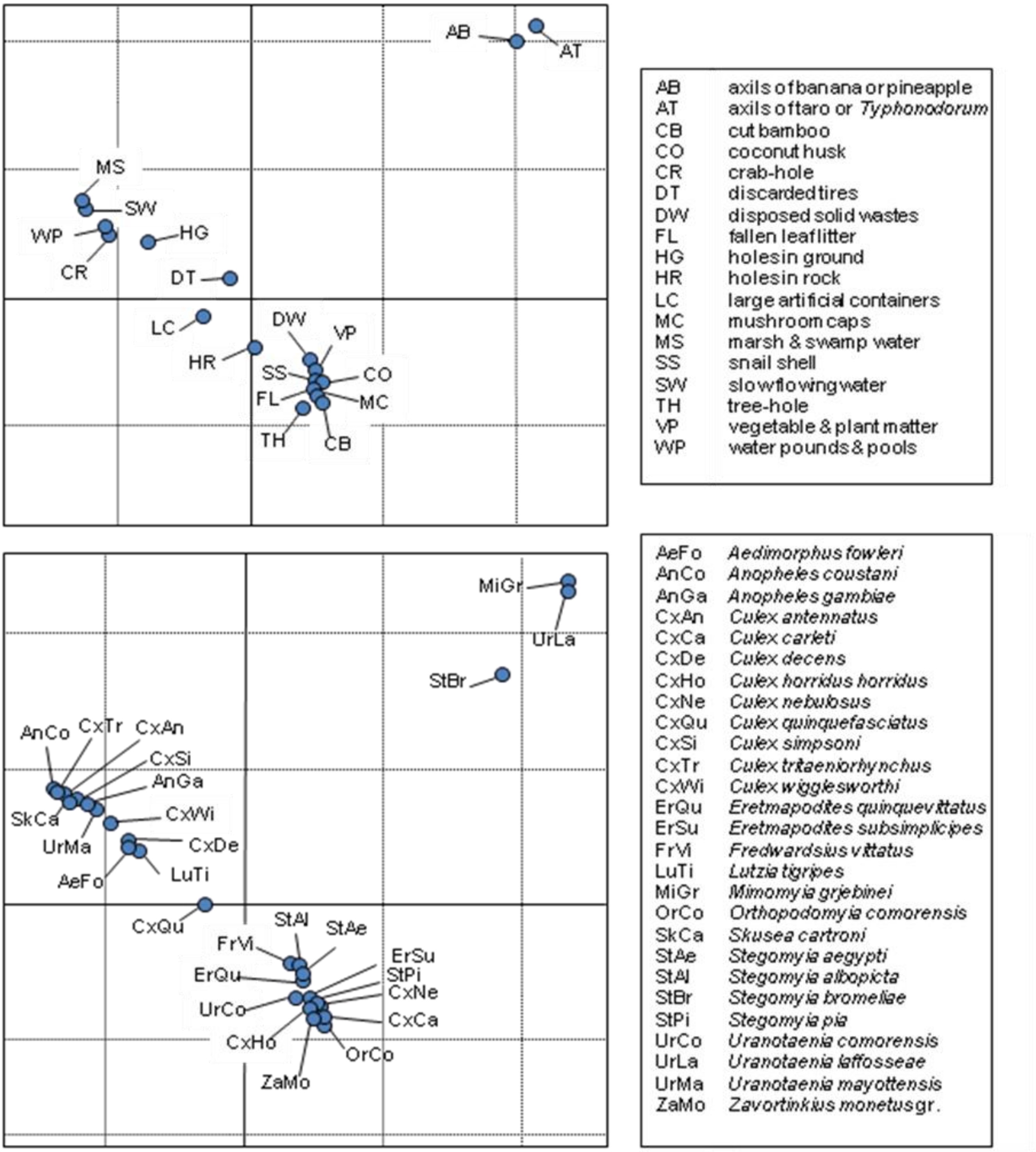

Figure A. Correspondence analysis for the 18 main types of larval habitats *vs*. the 27 main mosquito species. Here are represented the axis 1(horizontal) and 2 (vertical) that explained 43.9% (22.9% + 21.0%) of total variance; the scales are equal for the two graphs (side of each small quarter = 1).
